# Supplementary figures and images for: Constructing and validating a transferable epidemic risk index in data scarce environments using open data: A case study for dengue in the Philippines
Source: PLoS Negl Trop Dis. 2022 Feb 4;16(2):e0009262. doi: 10.1371/journal.pntd.0009262 (PMC8849499; doi:10.1371/journal.pntd.0009262)

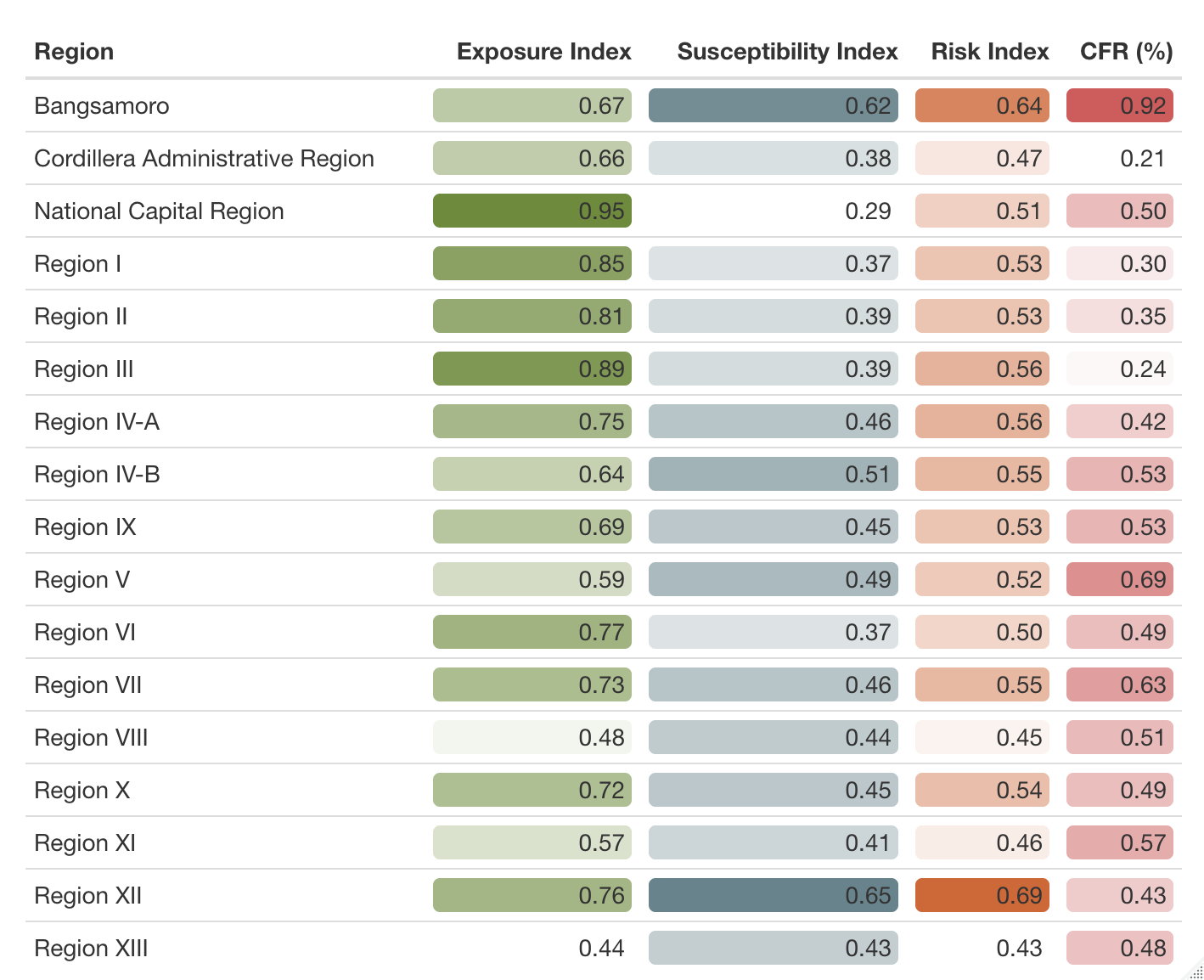

Supplement: S1 Fig — Absolute indices for individual dimensions, risk index and case fatality ratio. (PNG) [file pntd.0009262.s003.png]

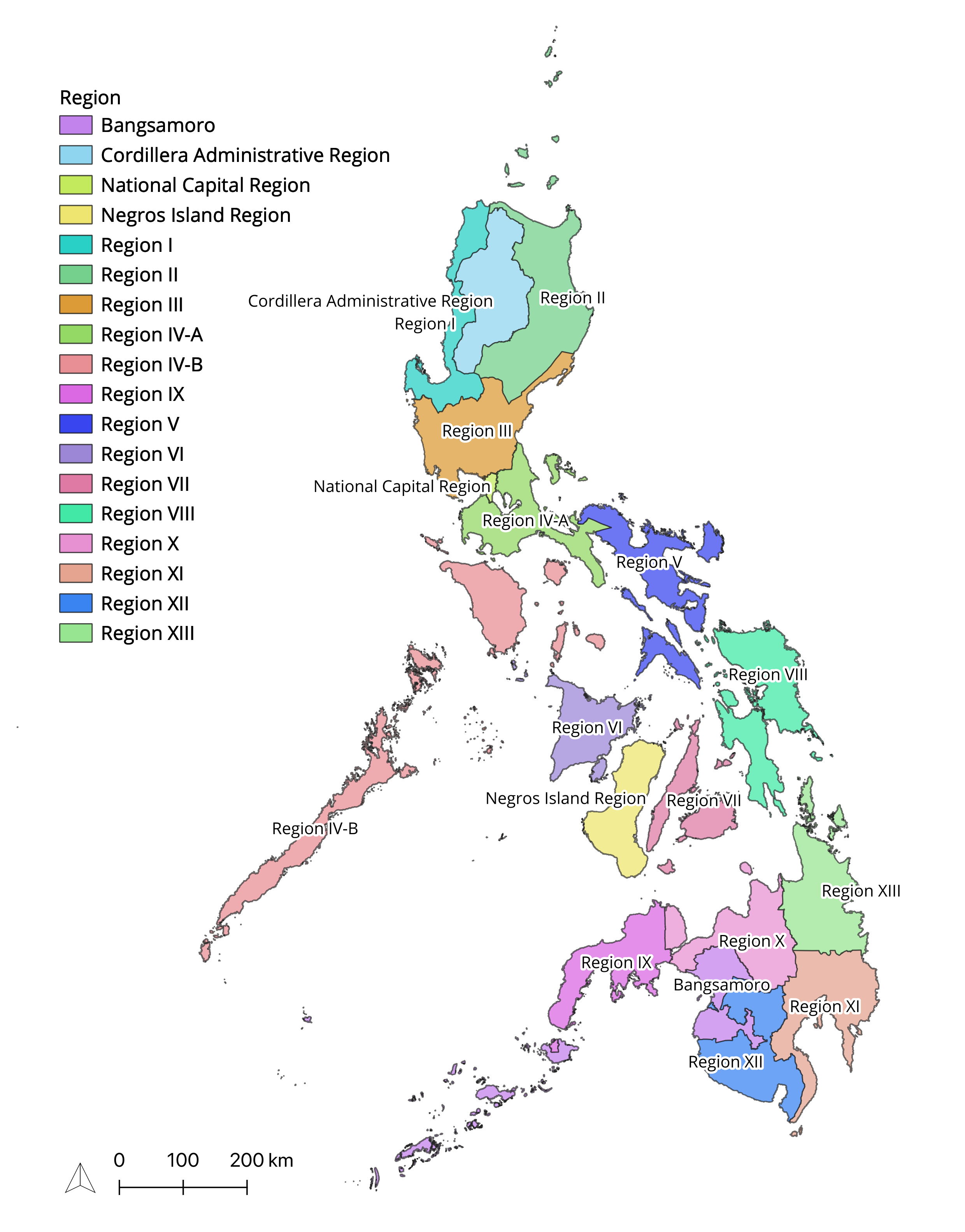

Supplement: S2 Fig — (PNG) [file pntd.0009262.s004.png]

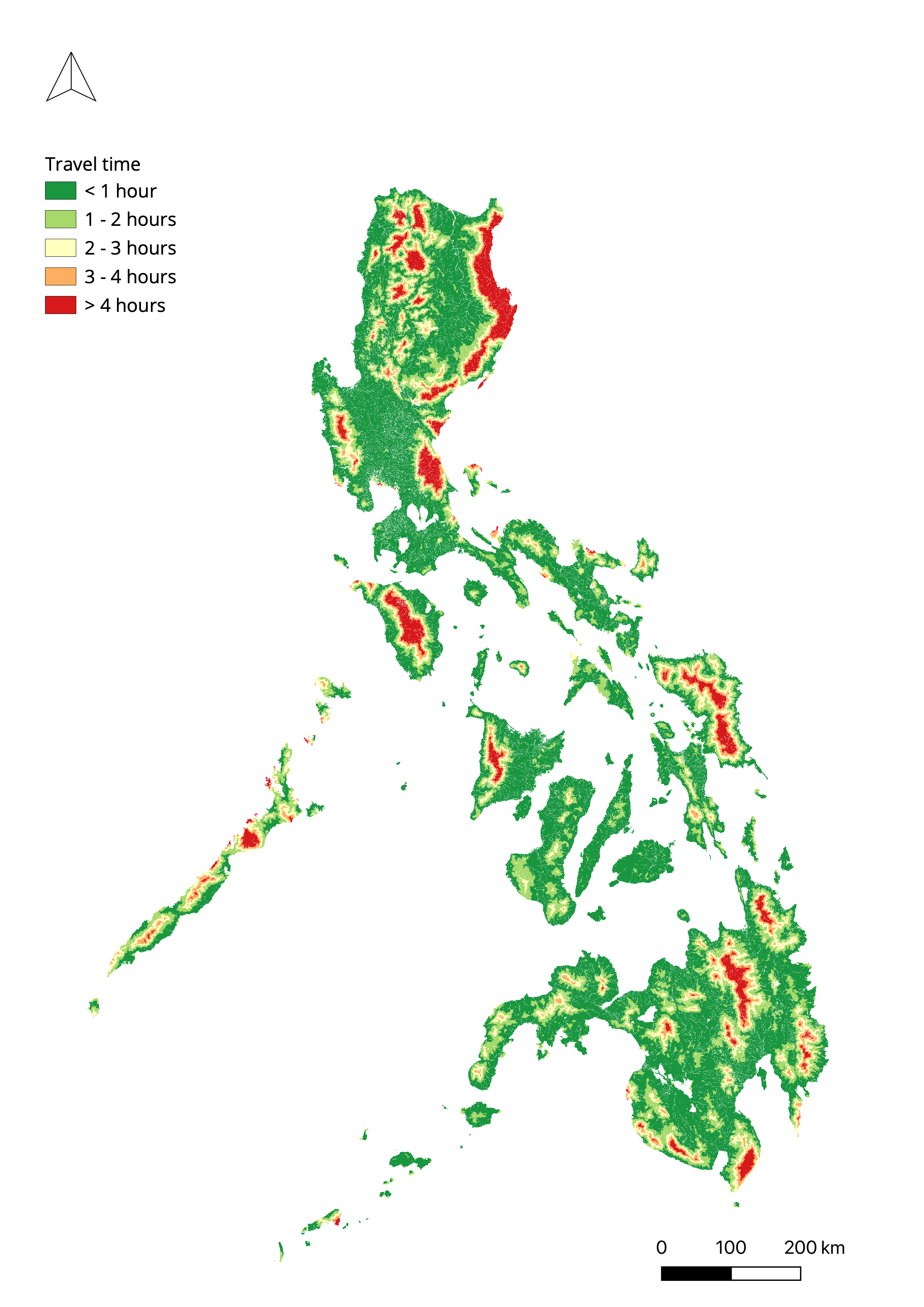

Supplement: S3 Fig — (PNG) [file pntd.0009262.s005.png]

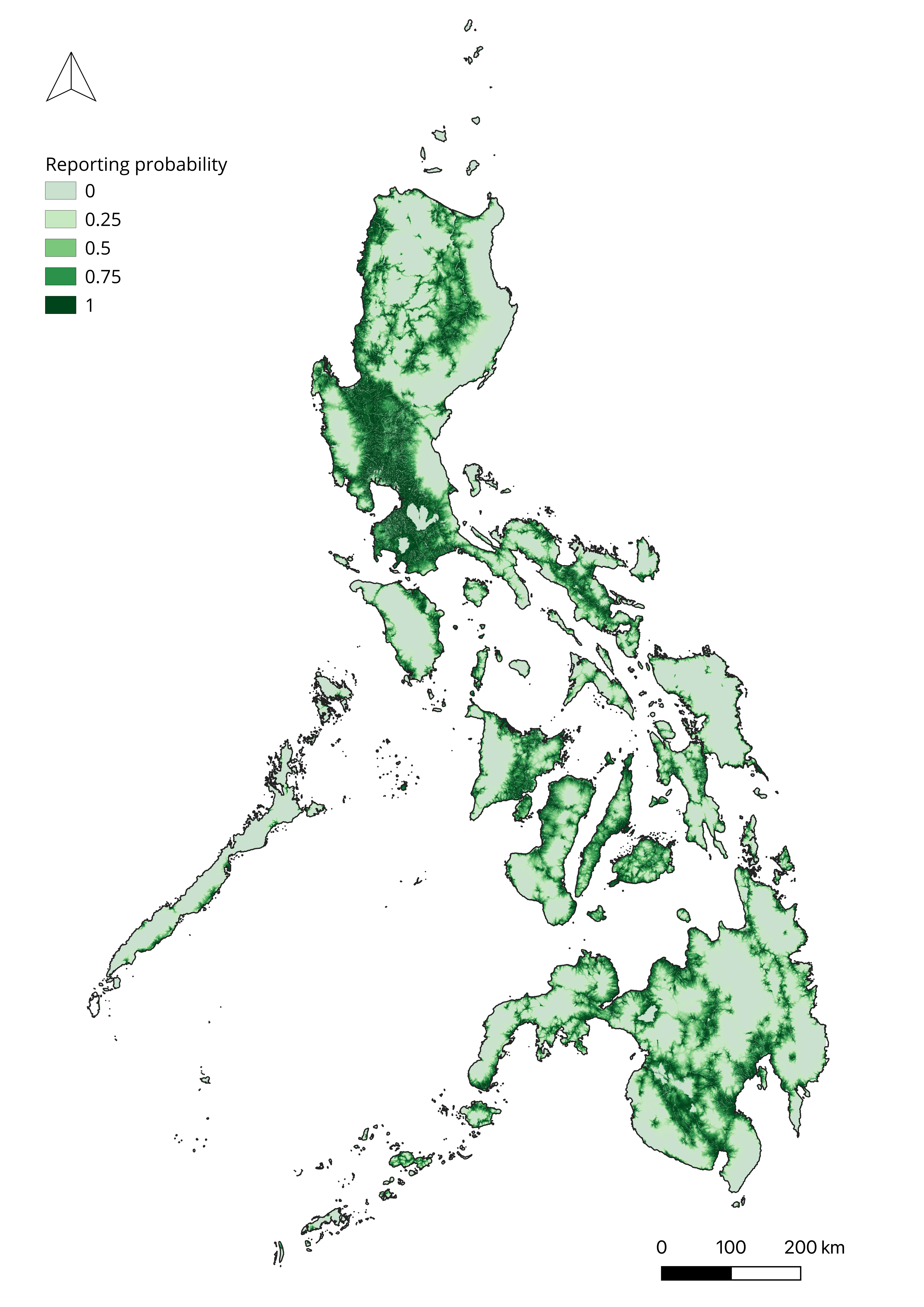

Supplement: S4 Fig — (PNG) [file pntd.0009262.s006.png]

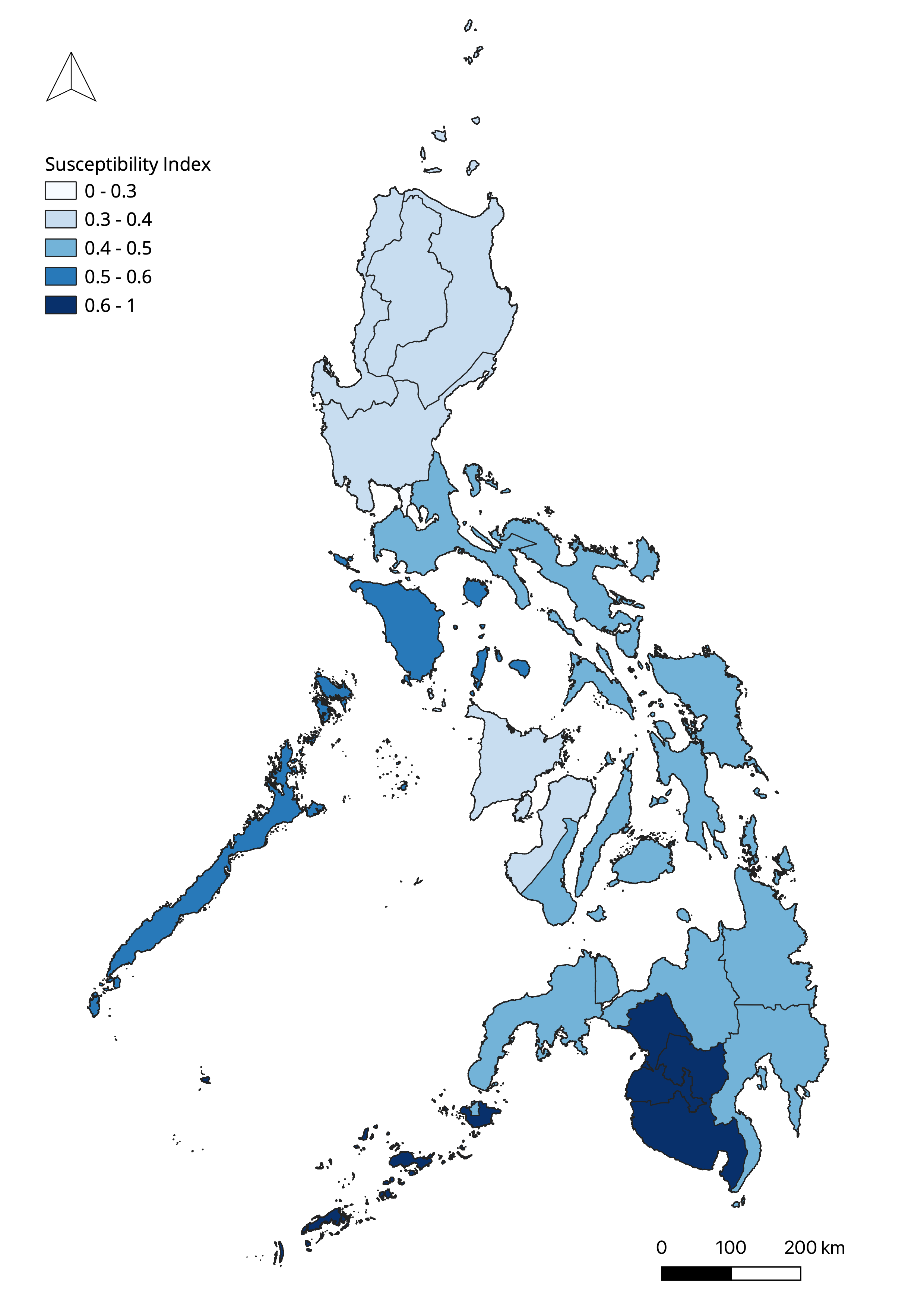

Supplement: S5 Fig — (PNG) [file pntd.0009262.s007.png]

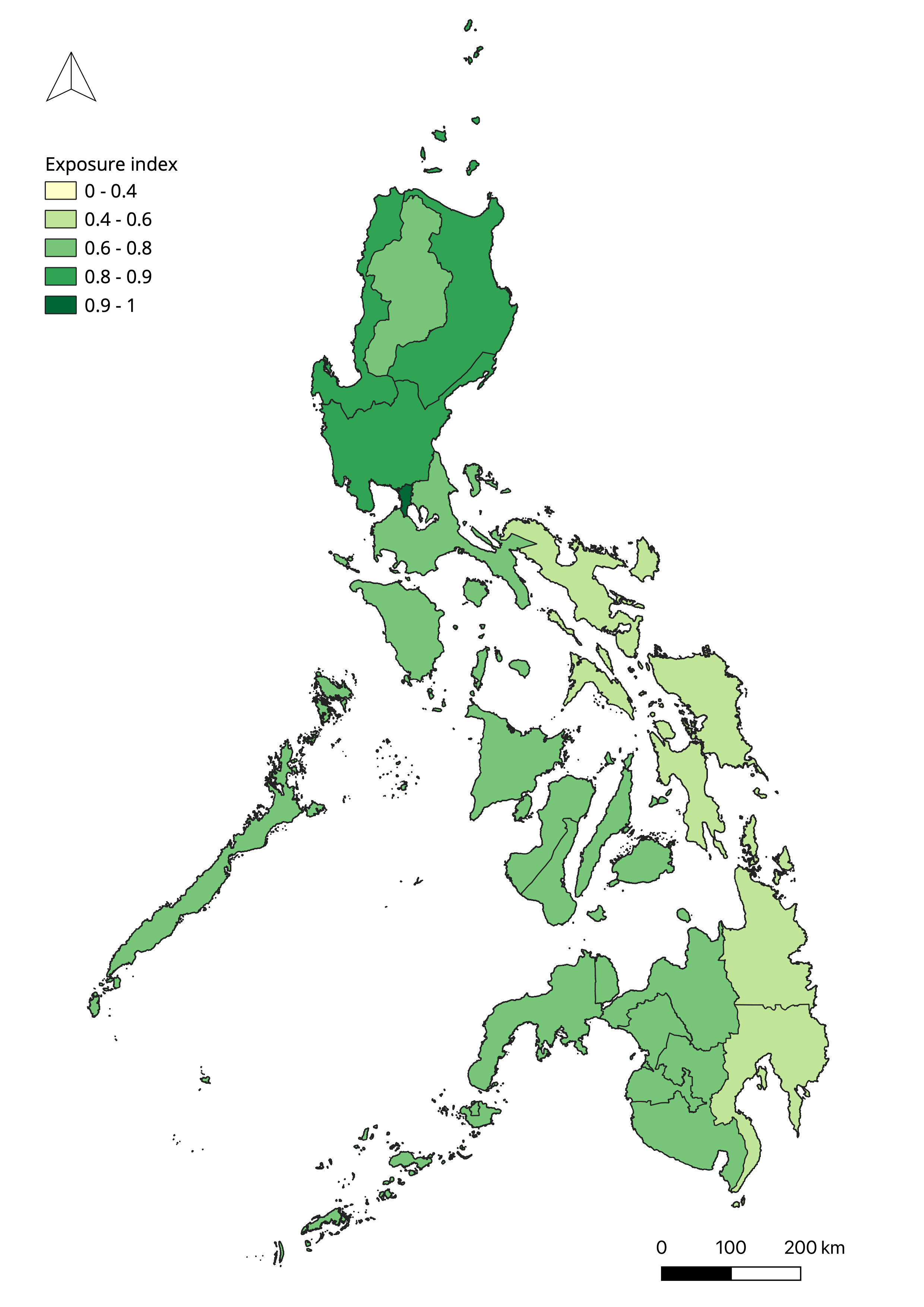

Supplement: S6 Fig — (PNG) [file pntd.0009262.s008.png]

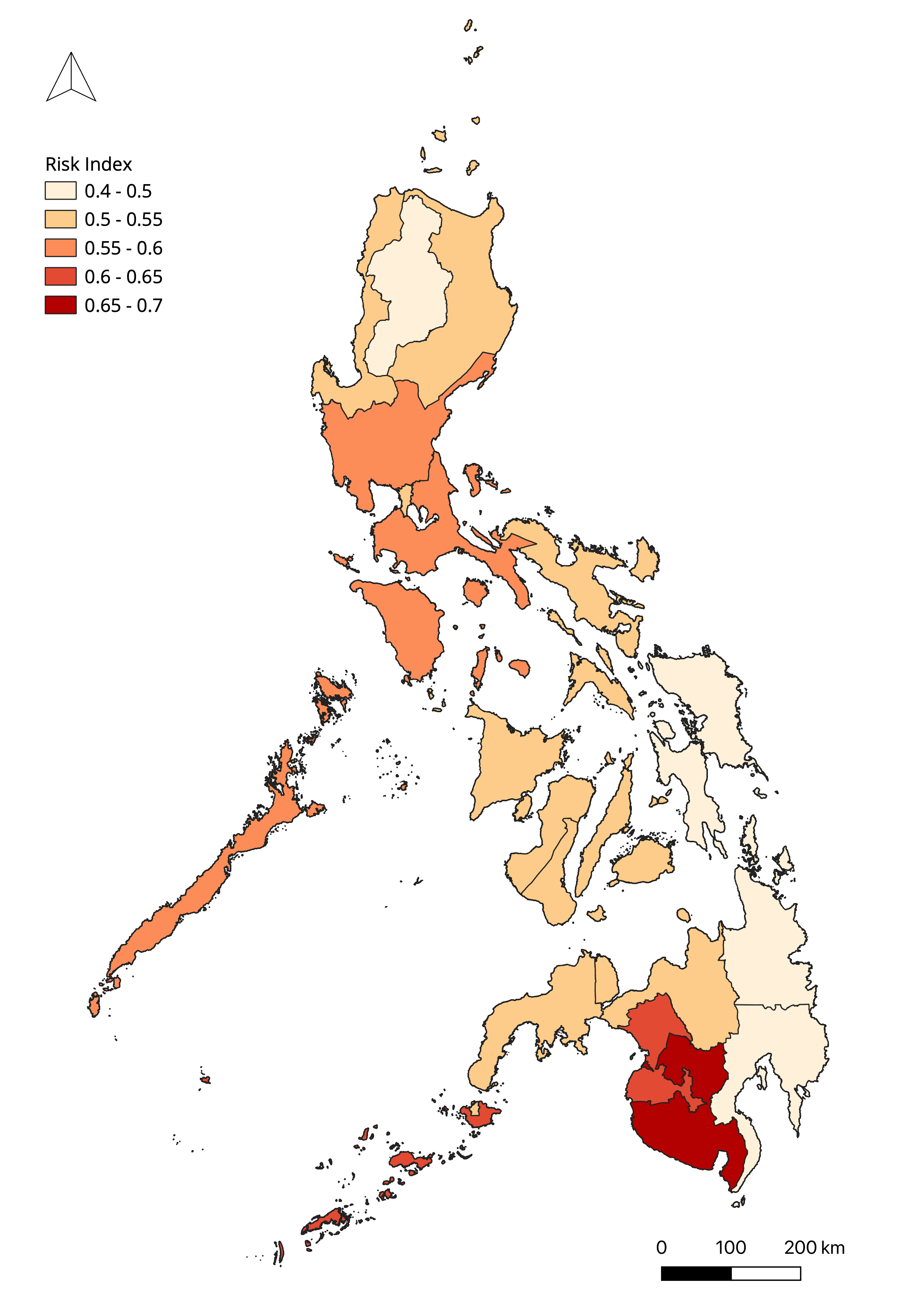

Supplement: S7 Fig — (PNG) [file pntd.0009262.s009.png]

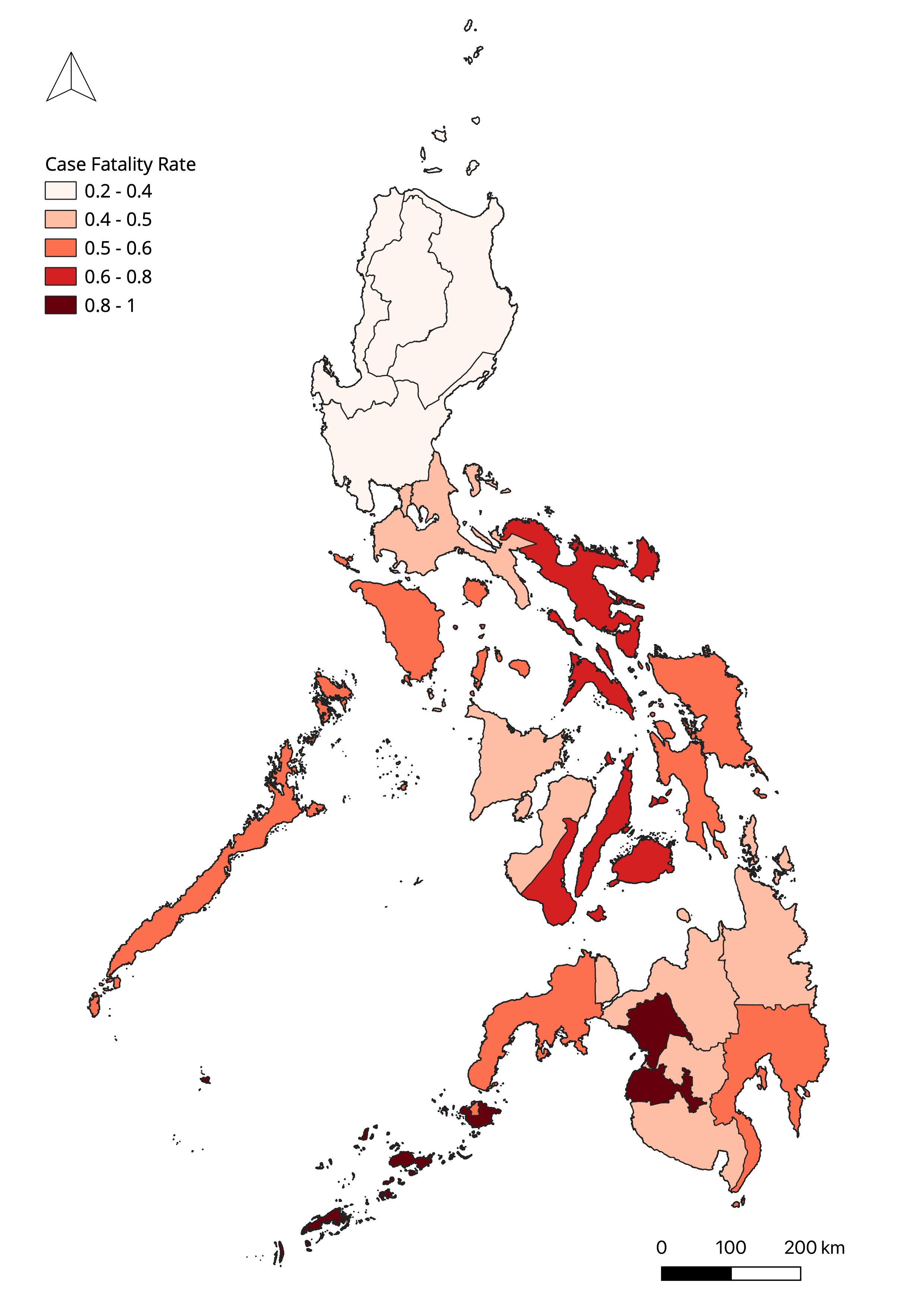

Supplement: S8 Fig — (PNG) [file pntd.0009262.s010.png]
